# Cancer epitope prediction tools and analysis pipelines in CEDAR

**DOI:** 10.1093/nar/gkag457

**Published:** 2026-05-12

**Authors:** Ibel Carri, Jason Greenbaum, Zhen Yan, Kevin Kim, Haeuk Kim, Ashmitaa Logandha Ramamoorthy Premlal, Daniel Marrama, Nina Blazeska, Hannah Carter, Ko-Han Lee, Timothy Sears, Morten Nielsen, Alessandro Sette, Bjoern Peters, Zeynep Koşaloğlu-Yalçın

**Affiliations:** Center for Vaccine Innovation, La Jolla Institute for Immunology, La Jolla, CA 92037, United States; Bioinformatics Core, La Jolla Institute for Immunology, La Jolla, CA 92037, United States; Bioinformatics Core, La Jolla Institute for Immunology, La Jolla, CA 92037, United States; Bioinformatics Core, La Jolla Institute for Immunology, La Jolla, CA 92037, United States; Bioinformatics Core, La Jolla Institute for Immunology, La Jolla, CA 92037, United States; Bioinformatics Core, La Jolla Institute for Immunology, La Jolla, CA 92037, United States; Center for Vaccine Innovation, La Jolla Institute for Immunology, La Jolla, CA 92037, United States; Center for Vaccine Innovation, La Jolla Institute for Immunology, La Jolla, CA 92037, United States; Department of Medicine, University of California, San Diego, La Jolla, CA 92093, United States; Department of Medicine, University of California, San Diego, La Jolla, CA 92093, United States; Department of Medicine, University of California, San Diego, La Jolla, CA 92093, United States; Department of Health Technology, Technical University of Denmark, Kgs, Lyngby DK-2800, Denmark; Center for Vaccine Innovation, La Jolla Institute for Immunology, La Jolla, CA 92037, United States; Department of Medicine, University of California, San Diego, La Jolla, CA 92093, United States; Center for Vaccine Innovation, La Jolla Institute for Immunology, La Jolla, CA 92037, United States; Department of Medicine, University of California, San Diego, La Jolla, CA 92093, United States; Center for Vaccine Innovation, La Jolla Institute for Immunology, La Jolla, CA 92037, United States

## Abstract

Accurate identification of immunogenic cancer epitopes remains a central challenge in immuno-oncology. The Cancer Epitope Database and Analysis Resource (CEDAR, https://cedar.iedb.org/) was developed to provide comprehensive curation of experimentally validated epitopes and to foster the development of computational tools tailored to the cancer context. Recently, we released a suite of cancer-specific tools and analysis pipelines as part of the https://nextgen-tools.iedb.org/ platform, enabling users to generate, evaluate, and prioritize candidate T cell epitopes in a modular framework. Here, we present the design and functionality of these tools, describe their core methodologies, provide guidance for their use, and illustrate how they can be integrated into end-to-end pipelines. We highlight applications in cancer immunology and personalized immunotherapy by presenting practical use cases.

## Graphical Abstract

**Graphical Abstract:**
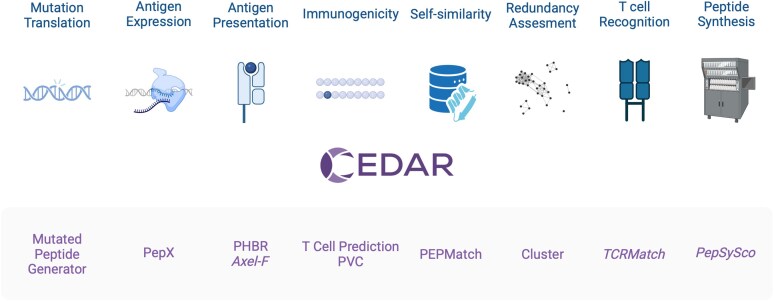

## Introduction

Adaptive immune responses directed against cancer epitopes can contribute to tumor control and response to immunotherapies. Cancer epitopes encompass mutated gene products, overexpressed proteins, cancer germline antigens, differentiation antigens, oncoviral proteins, and altered glycolipids and glycoproteins [1, 2]. Among these classes, mutation-derived neoepitopes have emerged as especially significant [3] as they are more likely to elicit robust T cell immune responses [4]. Next-generation sequencing (NGS) allows comprehensive identification of tumor mutations, yielding hundreds of potential neoepitope candidates in highly mutated cancers [4, 5]. Yet, only a small fraction of these candidates are immunogenic, meaning they are processed, presented, and recognized by T cells *in vivo* [6, 7]. Computational prediction tools can help to narrow down the set of candidate T cell epitopes for experimental validation and immunotherapy design.

Traditional epitope prediction algorithms developed in the context of infectious diseases have proven to be applicable to cancer immunology research [7, 8]. In recent years, new tools and pipelines have emerged that explicitly incorporate cancer-specific variables, including tumor heterogeneity, immune tolerance, and antigen abundance [9–13]. The development of such cancer-specific tools critically depends on the availability of curated datasets that include experimentally validated cancer epitopes. The Immune Epitope Database (IEDB, https://iedb.org/) has long served as a central resource for epitope data across infectious disease, allergy, and autoimmunity, providing the foundation for many of the prediction tools in use today [14, 15]. The Cancer Epitope Database and Analysis Resource (CEDAR, https://cedar.iedb.org/) was established to address the specific needs of the cancer immunology community, systematically cataloging immune responses to cancer antigens from thousands of studies published in the literature [14, 16, 17].

As part of CEDAR, novel cancer-specific tools have been developed and integrated with existing prediction algorithms within the IEDB Next-Generation Tools (NGT, https://nextgen-tools.iedb.org/) platform [18]. These tools enable cancer researchers to address key questions, such as identifying neoepitopes from somatic mutations, comparing the major histocompatibility complex (MHC) and human leukocyte antigen (HLA) binding of mutant and wild-type peptides, and estimating antigen abundance from publicly available expression datasets. Making the cancer-specific CEDAR tools available within the NGT provides a unified interface for epitope prediction, modular tool integration, and reproducible pipelines [18].

Here, we present the design and functionality of these tools, describe their core methodologies, provide guidance for their use, and illustrate how they can be integrated into end-to-end pipelines. We highlight key applications in cancer immunology research, such as the prioritization of shared, overexpressed epitopes, and neoepitope discovery from NGS data for personalized cancer immunotherapy, demonstrating how to effectively apply the tools in practice.

## Results

A central challenge in cancer immunology is that the computational tools required for epitope prediction and analysis are often inaccessible to researchers without programming expertise. Existing approaches are frequently command-line based, require local installation and configuration, or are embedded in supplementary materials that are difficult to reproduce. CEDAR tools address this by providing a unified, web-accessible platform that supports end-to-end cancer epitope analysis without requiring any programming experience.

Beyond accessibility, CEDAR tools offer a degree of modularity and cancer-specificity that is not available in existing platforms. While tools such as MuPeXI [19] and Seq2Neo [20] support neoepitope prediction from variant input, they operate as fixed workflows without support for modular pipeline construction or interactive filtering at intermediate steps. Component-level predictors such as HLAthena [13] and PRIME [21] address individual steps in the analysis but do not support end-to-end workflows. General-purpose frameworks such as Galaxy [22] can in principle support epitope prediction workflows through community-contributed tools but are not specialized for cancer immunology and do not natively include cancer-specific predictors. CEDAR tools address these gaps by integrating neopeptide generation from variant call files, expression-based filtering using tumor transcriptomic datasets, and cancer-specific immunogenicity scoring within a single modular interface, where users can combine tools into custom pipelines, apply filters at intermediate steps, and pass results directly between tools. The platform also supports programmatic access through a documented API, enabling advanced users to scale analyses beyond what is feasible in an interactive interface.

It is important to note that CEDAR tools does not currently prescribe a recommended ranking or prioritization strategy for epitope candidates; decisions about how to weigh and combine prediction scores remain with the user.

In the following sections, we provide a comprehensive overview of the CEDAR tools, focusing on its relevance to cancer epitope analysis. Some of these tools constitute traditional epitope prediction methods that have demonstrated broad applicability in cancer research [23], such as MHC binding and antigen presentation predictions, while others are specifically tailored to the cancer context. These tools can be utilized independently or as part of integrated pipeline workflows via the NGT platform. Novel tools being introduced here for the first time are described in detail, while previously published tools include citations for in-depth information.

### Mutated peptide generator

Mutated peptide generator (MPG) is designed to translate how somatic variants in DNA result in mutated peptide sequences. It supports single-nucleotide variants, multinucleotide variants, and insertions and deletions, and generates peptides that span the mutation site. The tool is designed to identify candidate neoepitopes from tumor variant datasets and can be integrated into larger prediction pipelines. By preserving alignment between wild type and mutant peptides, MPG facilitates downstream side-by-side comparison of their immunological properties.

MPG accepts variant call format (VCF) files as input and uses SnpEff [24] for variant annotation to determine coding consequences, affected transcripts, and resulting amino acid changes. The tool accepts pre-annotated VCFs or can internally perform SnpEff annotation. To ensure consistent and accurate peptide generation, users are encouraged to normalize and decompose their VCFs [25]. Additional arguments include peptide length, mutation position within the peptide, and overlap length for frameshift-derived sequences.

The output consists of three structured tables that capture the mapping from variant to peptide at varying levels of resolution. The variant table provides one row per variant with detailed annotations. The peptide table includes one row per variant–transcript pair, listing all derived peptides and reflecting transcript-specific effects. The unique peptide table collapses redundant sequences across transcripts and selects a representative transcript for each unique peptide. All outputs include metadata such as genomic coordinates, coding context, mutation position, and peptide sequences, supporting their integration into downstream antigen presentation and immunogenicity prediction tools.

### Peptide expression annotation

The relevance of antigen abundance in epitope discovery is well documented; we and others have previously shown that it is a predictor of neoantigen immunogenicity [11, 26]. Peptide expression annotation (PepX) [27] enables peptide-level expression annotation by estimating the abundance of source antigens using public transcriptomic datasets. It is particularly valuable when patient-specific RNA-Seq data are not available, as it provides context-specific expression estimates from well-established reference cohorts.

The tool accepts peptide sequences as input and identifies all protein isoforms from which each peptide may originate. Users can select from multiple public RNA-Seq datasets, including The Cancer Genome Atlas (TCGA) [28], Genotype-Tissue Expression (GTEx) [28, 29], Human Protein Atlas (HPA) [30], and the Cancer Cell Line Encyclopedia (CCLE) [31], and expression can be quantified at gene or transcript level. Transcript-level quantification offers higher resolution and outperforms gene-level estimates in distinguishing eluted ligands from decoy peptides across multiple validation datasets (mean AUC = 0.816, [27]). A key limitation is that these datasets provide population-level expression summaries rather than individual patient values; however, TCGA median expression values correlate strongly with patient-matched data (Spearman *r* = 0.82), making them an adequate surrogate when patient-specific RNA-Seq is unavailable [27].

### T cell prediction—class I

The IEDB T cell prediction—class I tools provide predictions for MHC class I antigen processing, presentation, and CD8 T cell recognition. These predictions serve as foundational components for epitope prioritization workflows across cancer and other disease contexts. The tool supports three prediction types, which have been described in detail elsewhere: (i) MHC class I binding/elution predictions, including NetMHCpan EL (eluted ligand), NetMHCpan BA (binding affinity) [32], MHCflurry [33], and IEDB Consensus [34], among others; (ii) Immunogenicity predictions [35]; and (iii) Antigen processing predictions using the IEDB Consensus [34], NetCTL [36], NetCTLpan [37], and NetChop [38].

Based on independent benchmarks [39], CEDAR currently recommends the NetMHCpan model as the default for MHC class I epitope predictions. NetMHCpan 4.1 EL has demonstrated state-of-the-art performance in identifying both eluted ligands (mean AUC = 0.949) and CD8 + T cell epitopes (AUC = 0.989) [32].

NetMHCpan predictions are reported as percentile ranks, which represents the predicted binding strength of a peptide relative to a background distribution of random natural peptides. This normalization avoids biases introduced by MHC alleles with inherently higher or lower predicted affinities. Peptides with lower percentile ranks are more likely to be presented, with strong binders having scores ≤0.5 and weak binders <2.0 [40].

### T cell prediction—class II

Similar to the T cell prediction—class I tools, the T cell prediction—class II tools provide predictions of MHC class II antigen processing, presentation, and CD4 T cell recognition. These tools are designed to identify peptides likely to be presented on the cell surface of antigen presenting cells (APCs), enabling the filtering of candidate epitopes. The tool supports three prediction types, which have been described in detail elsewhere. (i) MHC class II binding/elution predictions with NetMHCIIpan EL (recommended) and BA models [41], the IEDB consensus [34], and others; (ii) Immunogenicity predictions with the CD4Episcore model [42]; and (iii) Antigen processing predictions with MHCII-NP [43].

Currently, CEDAR recommends the NetMHCIIpan 4.1 EL [44] method for MHC class II epitope prediction based on independent benchmarks, where it showed increased performance in both antigen presentation (AUC= 0.835) [45] and CD4 neoepitope predictions (AUC = 0.838) [44].

NetMHCIIpan predictions are reported as percentile ranks (see previous section); lower percentile ranks indicate higher likelihood of presentation, with strong binders scoring ≤2 and weak binders <10 [44].

### Peptide variant comparison

The peptide variant comparison (PVC) tool enables systematic evaluation of somatic mutations by comparing mutant and wild-type peptides side-by-side across multiple immunological prediction models (listed in the two previous sections). This approach is designed to assess whether a mutation enhances or reduces peptide presentation and immunogenicity, and is particularly relevant in neoepitope prioritization workflows.

As input, PVC accepts pairs of mutant and wild-type peptides, and MHC alleles. The tool supports three prediction types: (i) MHC class I and II antigen presentation using algorithms such as MHCFlurry and NetMHCIIpan; (ii) general immunogenicity predictions [35, 42]; and (iii) neoepitope immunogenicity predictions using the ICORE-based prediction of neo-epitope immunogenicity (ICERFIRE) model [9]. Notably, ICERFIRE has demonstrated superior predictive performance in independent neoepitope datasets (AUC = 0.726), outperforming state-of-the-art methods.

PVC outputs include side-by-side prediction scores for each peptide pair across all selected models, as well as the computed differences in scores between mutant and wild-type sequences. While the specific interpretation of these values depends on the algorithm selected, as a general rule, lower IC_50_​ values, lower percentile ranks, and higher predictive scores indicate a higher likelihood of antigen presentation or immunogenicity. Visualizations are generated as scatterplots, allowing users to assess shifts in binding or immunogenicity in a graphical format. These results can be used to rank mutations and identify candidates for further experimental validation.

### PEPMatch

PEPMatch [46] enables rapid identification of sequence-similar peptides across curated reference proteomes, supporting the evaluation of potential cross-reactivity in epitope discovery workflows.

The tool accepts a list of input peptides and allows users to define the maximum number of amino acid substitutions permitted in the search. The output consists of a tabular summary with one row per input–match pair, reporting the matched sequence and associated information. Peptides with no matches can be retained in the output. These results enable the exclusion of peptides highly similar to self-antigens, prioritizing targets with better specificity for experimental validation.

PEPMatch is built on a deterministic *k*-mer mapping algorithm that preprocesses the reference proteome prior to searching. Benchmarking against established datasets demonstrated that general-purpose alignment tools such as BLAST [47] recover only 58% of true matches in a neoepitope dataset of 620 15-mers searched against the human proteome with up to 3 mismatches, while PEPMatch achieves 100% recall on the same dataset with faster search times [46].

### Patient harmonic-mean best rank

Patient harmonic-mean best rank (PHBR) [48, 49] summarizes peptide–MHC binding predictions across a patient’s HLA genotype to identify candidate mutations with high immunogenic potential. Rather than relying on binding strength to a single HLA allele, PHBR captures the overall likelihood of presentation by aggregating predictions across all relevant alleles using a harmonic mean. The harmonic mean is heavily influenced by the lowest score, effectively reflecting the presentation potential of mutations that result in highly presented peptides. This metric has been shown to correlate with immune responsiveness, particularly in the context of checkpoint blockade therapy, and is valuable for prioritizing candidate neoepitopes in personalized cancer immunotherapy studies. The performance of PHBR antigen presentation in MHC class I is AUC = 0.69 [48], and AUC = 0.75 for MHC class II [49].

### Clustering

The Clustering tool [50] enables the generation of biologically meaningful consensus sequences and redundancy reduction in immune epitope datasets by grouping similar sequences. The tool’s output includes a cluster assignment for each input peptide, and a representative or consensus sequence per cluster. The representative set generated by Clustering has been validated across multiple immune datasets, including MHC Class I and II ligands and epitopes, achieving an average redundancy reduction of 56% of the total input sequences [50]. By reducing redundancy, the tool simplifies downstream analysis and helps prioritize nonredundant peptide sets for visualization or experimental testing. The generation of representative sequences is especially useful for vaccine design and for collapsing overlapping peptides that arise from sliding window predictions, alternative transcripts, or post-translational variants.

### Additional tools for cancer epitope prediction workflows

Several tools remain available on the original IEDB Analysis Resource (AR, https://tools.iedb.org/) site and continue to support cancer epitope prediction workflows [51]. These include tools for TCR specificity prediction (TCRMatch), peptide synthesis prediction (PepSySco), and MHC binding predictions integrated with antigen abundance (AXEL-F) [11, 52, 53]. These tools will be incrementally integrated into the NGT platform over the coming years. This planned migration will ensure that the full breadth of IEDB’s analytical capabilities is accessible through a unified interface that supports end-to-end pipeline construction.

### Pipeline integration

In cancer research, the true power of individual tools lies in their integration into comprehensive analysis pipelines. NGT supports the construction of such pipelines, enabling users to combine multiple tools into structured, reproducible workflows. Pipelines allow the sequential execution of analyses in which the output of one tool serves as the input for the next. At each stage, intermediate results can be reviewed and filtered, permitting the propagation of selected candidates downstream.

This functionality is designed to facilitate the systematic evaluation of large candidate sets and supports common use cases in cancer epitope discovery. The pipeline interface includes a visual sidebar that maps the analysis steps and their parameters. Users may save, revisit, and share pipelines via stable URLs, either including input and output data or preserving only the pipeline specifications. Programmatic access for automated or large-scale analyses is available through a documented API (refer to https://nextgen-tools.iedb.org/docs/api/index.html).

The following case scenarios illustrate end-to-end workflows that cannot be fully reproduced using any single existing platform, demonstrating the practical value of CEDAR tools’ integrated and modular design.

### Case scenario I: expression-based filtering of candidate tumor-associated antigens in NSCLC

A translational research team discovered a set of tumor–associated proteins in an in-house nonsmall cell lung cancer (NSCLC) cohort and is now evaluating their immunogenic potential. Although these proteins appeared overexpressed in the initial small dataset, the team seeks to confirm expression patterns and tissue specificity in larger, independent cohorts, and select peptides that might be suitable for inclusion in a therapeutic vaccine.

The researchers used PepX to cross-reference their candidate peptides against the TCGA-LUAD (Lung Adenocarcinoma) dataset. To ensure that only biologically relevant candidates were prioritized, a minimum expression threshold (e.g. 1 TPM) was applied to the “Total Transcript TPM” values. This filtering step ensures that the vaccine targets are derived from genes with confirmed expression in the specific cancer type of interest, minimizing the risk of targeting low-abundance antigens that may escape immune detection.

The next phase of the pipeline integrates MHC class I antigen presentation predictions to assess whether these expressed peptides are likely to form a complex with MHC molecules and be subsequently presented on the tumor cell surface. To ensure the findings are applicable to a diverse patient population, the researchers employed the 27-allele panel designed to cover >97% of the global population [54].

Employing the recommended NetMHCpan 4.1 EL model, the team evaluated the “percentile rank” for each candidate. The percentile rank neutralizes inherent biases in MHC-allele binding, allowing the usage of unique filtering thresholds across multiple MHC molecules. By applying established biological thresholds (percentile rank <2), the team filtered for peptides with high likelihood of extracellular presentation.

**Figure 1.**
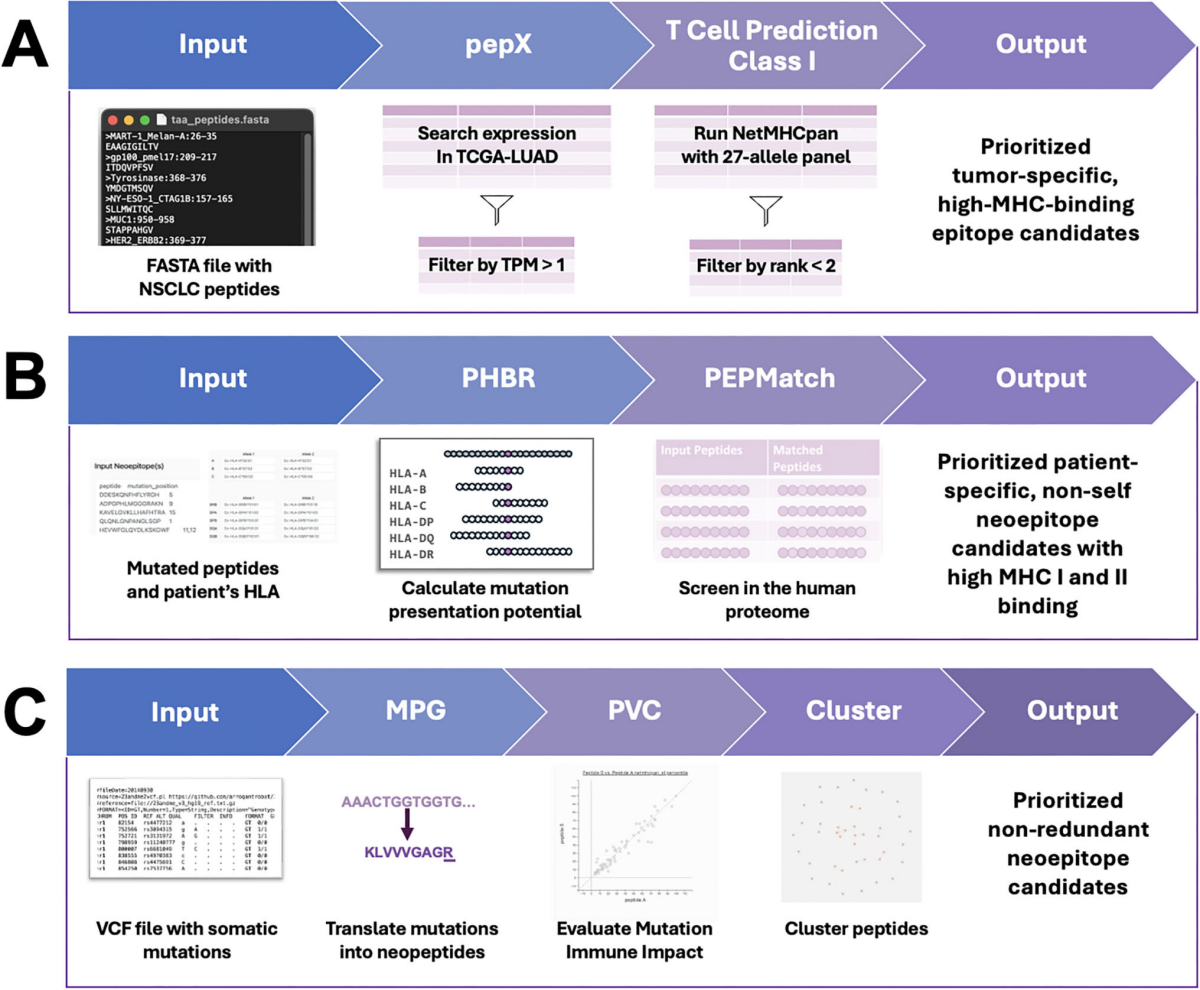
Schematic representation of cancer epitope analysis workflows. (**A**) Case scenario I: prioritizing tumor-associated antigens in NSCLC through expression-based filtering and MHC presentation scoring. (**B**) Case scenario II: personalized neoepitope discovery in glioblastoma using patient-specific HLA ranking (PHBR) and proteome-wide safety screening. (**C**) Case scenario III: analysis of shared RAS hotspot mutations via variant translation, immunogenicity comparison, and sequence redundancy clustering.

This integrated immunoinformatics pipeline prioritizes peptides that satisfy two essential biological requirements: high malignancy-specific expression and a high probability of MHC-mediated antigen presentation. Reducing a large candidate set to a curated panel of high-confidence targets increases the feasibility of experimental validation (Fig. 1A). A detailed step-by-step description of this pipeline is provided in Supplementary Fig. S1.

### Case scenario II: neoepitope discovery for personalized immunotherapy in glioblastoma

A neuro-oncology team is developing a personalized mRNA vaccine for a glioblastoma patient. They have already performed variant calling based on whole-exome sequencing data of paired tumor and normal samples. Their goal is to prioritize highly expressed mutations subject to T cell recognition.

To capture the cumulative likelihood of a mutation being presented across the patient’s specific HLA genotype, the team utilized the PHBR tool. Unlike methods that rely on a single “best-binding” allele, PHBR provides a biologically representative score by integrating the presentation potential across all of the patient’s MHC class I and class II alleles. By selecting the NetMHCpan 4.1 EL and NetMHCIIpan 4.3 EL models, the team leveraged state-of-the-art methods to predict which somatic mutations are most likely to be processed and presented on the cell surface. Mutations were then ranked by their PHBR scores, allowing the researchers to focus on the top-tier candidate mutations with the highest statistical probability of T cell exposure.

A critical requirement for any personalized immunotherapy is the exclusion of “self-peptides” to avoid the risk of off-target autoimmunity and to ensure the vaccine targets sequences not subject to central immune tolerance. To achieve this, the prioritized candidates were passed to PEPMatch for screening against the entire human reference proteome. By setting a strict threshold of zero mismatches, the team successfully identified and excluded any mutant-derived peptides that shared exact sequences with normal human proteins.

This modular pipeline allows the team to prioritize patient-specific glioblastoma neopeptides with high MHC class I and II binding potential and that are not present in the human proteome, yielding a refined set of safe candidate neoepitopes for therapeutic development (Fig. 1B). A detailed step-by-step description of this pipeline is provided in Supplementary Fig. S2.

### Case scenario III: evaluation of shared RAS neoepitopes across tumor types

A collaborative team is investigating shared CD8+ T cell neoepitopes arising from common KRAS, NRAS, and HRAS hotspot mutations across multiple cancer types, including pancreatic, colorectal, and lung adenocarcinomas. The aim is to identify widely presented, nonredundant, and low-risk neoepitopes for pan-cancer immunotherapy.

Based on a compiled set of frequent mutations in the KRAS, NRAS, and HRAS genes, the team utilized the MPG to generate 9-mer peptides, the predominant length for MHC class I ligands. The researchers prioritized two primary mechanisms of neoantigenicity by strategically positioning the mutated residues: first, at the C-terminal MHC anchor position, to identify neopeptides that gain immunogenicity through novel MHC stabilization (thereby bypassing tolerance to the wild-type sequence); and second, within the TCR contact site (position 5) to target epitopes capable of engaging T cell repertoires that escaped central thymic deletion.

Following this, the pipeline integrated the PVC, enabling a multiparametric evaluation of candidates through the ICERFIRE model. ICERFIRE ranks peptides by modeling pan-cancer transcriptional prevalence (via the TCGA pan-cancer dataset), with antigen presentation likelihood and T cell recognition propensity. To guarantee clinical relevance across a diverse global population, a 27-allele panel [54] was utilized.

Recognizing that hotspot mutations in highly conserved RAS isoforms often yield peptides with high sequence similarity, the team employed the Cluster module to eliminate redundancy. The cluster method grouped overlapping or highly similar epitope candidates and provided unique representative sequences per cluster. This approach maximizes the potential breadth of the immune responses while preventing the inclusion of redundant peptides.

This pan-cancer discovery pipeline helped the team to identify nonredundant public neoepitope candidates that are broadly presentable for further preclinical development (Fig. 1C). A detailed step-by-step description of this pipeline is provided in Supplementary Fig. S3.

### Conclusion and discussion

Accurate and efficient identification of cancer T cell epitopes remains a central challenge in immuno-oncology. While foundational tools for MHC binding prediction have long served the broader immunology community, recent advances in cancer immunogenomics have created a demand for methods that can accommodate the biological and clinical features relevant to tumor biology, including somatic mutations, tumor-specific antigen expression, patient HLA diversity, and tolerance mechanisms, all of which impact epitope presentation and immunogenicity.

CEDAR tools address this by combining well-validated MHC binding predictors with newly developed cancer-specific tools, allowing users to move systematically from genomic data to prioritized epitope candidates.The development and refinement of these tools have been guided by feedback from the cancer immunology community, and iterative improvements in functionality and output interpretation have ensured that the tools remain aligned with experimental workflows and translational applications.

Integrated within the Next-Generation IEDB Tools platform (NGT), CEDAR tools can be used independently or assembled into custom pipelines that mirror the biological steps of antigen processing and immune recognition. The pipeline builder supports filtering at each step, data retention, and reproducibility through shareable links and programmatic access via API, enabling both novice and advanced users to construct analyses tailored to diverse research questions and datasets.

A deliberate design choice of the current CEDAR tools platform is the absence of a prescribed ranking or prioritization algorithm for epitope candidates. Rather than directing users toward a single prioritization strategy, the platform provides transparent access to individual prediction tools that can be combined and filtered according to the needs of a given study. This reflects the current state of the field: the landscape of cancer epitope prioritization methods is evolving rapidly, with new combinations of features and scoring strategies published on a regular basis [9, 10, 55–57]. No single approach has yet demonstrated robust generalization across cancer types [58], HLA backgrounds, and experimental contexts, and direct comparison across methods remains difficult due to differences in training data, evaluation datasets, and underlying research objectives. We are actively working to address this through systematic benchmarking of epitope prediction and prioritization tools using curated CEDAR datasets. This effort builds directly on the live benchmarking infrastructure we have previously established for MHC class I and class II binding predictions in the IEDB, in which automated pipelines generate predictions on new datasets immediately prior to their public release and performance metrics are updated on a continuous basis [39, 45]. Extending this framework to cancer-specific prioritization pipelines will enable regularly updated, evidence-based recommendations to guide both tool developers and end users.

Several platform limitations remain to be addressed in future releases. Some analytical tools relevant to cancer epitope research remain available only through the original IEDB Analysis Resource (AR, https://tools.iedb.org/) and have not yet been fully integrated into NGT. Tools such as AXEL-F, TCRMatch, and PepSySco are very relevant for cancer research as they cover additional steps in a typical cancer epitope analysis pipeline [11, 52, 53]. The planned incremental migration of these tools will help consolidate their functionality within a unified framework and make them available to include in end-to-end pipelines. Furthermore, for tools such as PepX, expression annotation currently relies on publicly available datasets, including TCGA, GTEx, and CCLE. While these provide adequate surrogates, patient-matched transcriptomic data remains preferable when available, particularly for personalized immunotherapy applications.

Beyond platform-specific limitations, users should also be aware of the inherent challenges in computational epitope prediction, which apply broadly across tools. MHC binding prediction, while well-validated and extensively benchmarked, is necessary but not sufficient for identifying immunogenic epitopes. Only a small fraction of predicted neo-peptides are ultimately recognized by T cells *in vivo*, and the gap between predicted binding and experimental immunogenicity remains a central challenge in the field [6, 10, 55, 59]. Factors including antigen processing efficiency, T cell repertoire diversity, central and peripheral tolerance, and the immunosuppressive tumor microenvironment all influence whether a predicted epitope elicits a functional immune response, and these are not fully captured by current computational models. Translational application of these tools introduces additional complexity because many predicted epitopes bind MHC molecules but fail to trigger T cell responses. One explanation is that current pipelines do not fully account for mutation clonality, antigen processing efficiency, and TCR recognition, all of which are critical determinants of true immunogenicity [60]. Furthermore, the neoepitope landscape is not static: as tumors evolve, immune editing can lead to the loss of immunogenic clones and shifts in the TCR repertoire. Current tools do not fully capture these dynamic processes, so predictions made at a single time point may not reflect a tumor’s immunogenic potential at the time of treatment [60].

While this manuscript focuses primarily on T cell-mediated immunity, B cell-mediated immune responses have also been demonstrated to play a key role in antitumor immunity [61]. A key feature of CEDAR tools is that it provides access to B cell epitope prediction tools, making it one of the few cancer epitope platforms to support both humoral and cellular immune response analysis within a unified framework. A more comprehensive description of these tools and their application in cancer research will be the focus of future work.

CEDAR tools offer a flexible and scalable computational foundation for cancer epitope analysis. By integrating genomic and immunological features into modular, accessible workflows, the platform supports both exploratory and hypothesis-driven research and is designed to grow alongside the rapidly evolving field of cancer immunotherapy.

## Supplementary Material

gkag457_Supplemental_File

## Data Availability

The CEDAR tools (web, API, and standalone) are freely available at https://nextgen-tools.iedb.org.
