# Supplementary material for: Cancer epitope prediction tools and analysis pipelines in CEDAR
Source: Nucleic Acids Res. 2026 May 12;54(W1):W13–21. doi: 10.1093/nar/gkag457 (PMC13355047; doi:10.1093/nar/gkag457)
Supplement: gkag457_Supplemental_File [file gkag457_supplemental_file.pdf]

Supplementary Figures

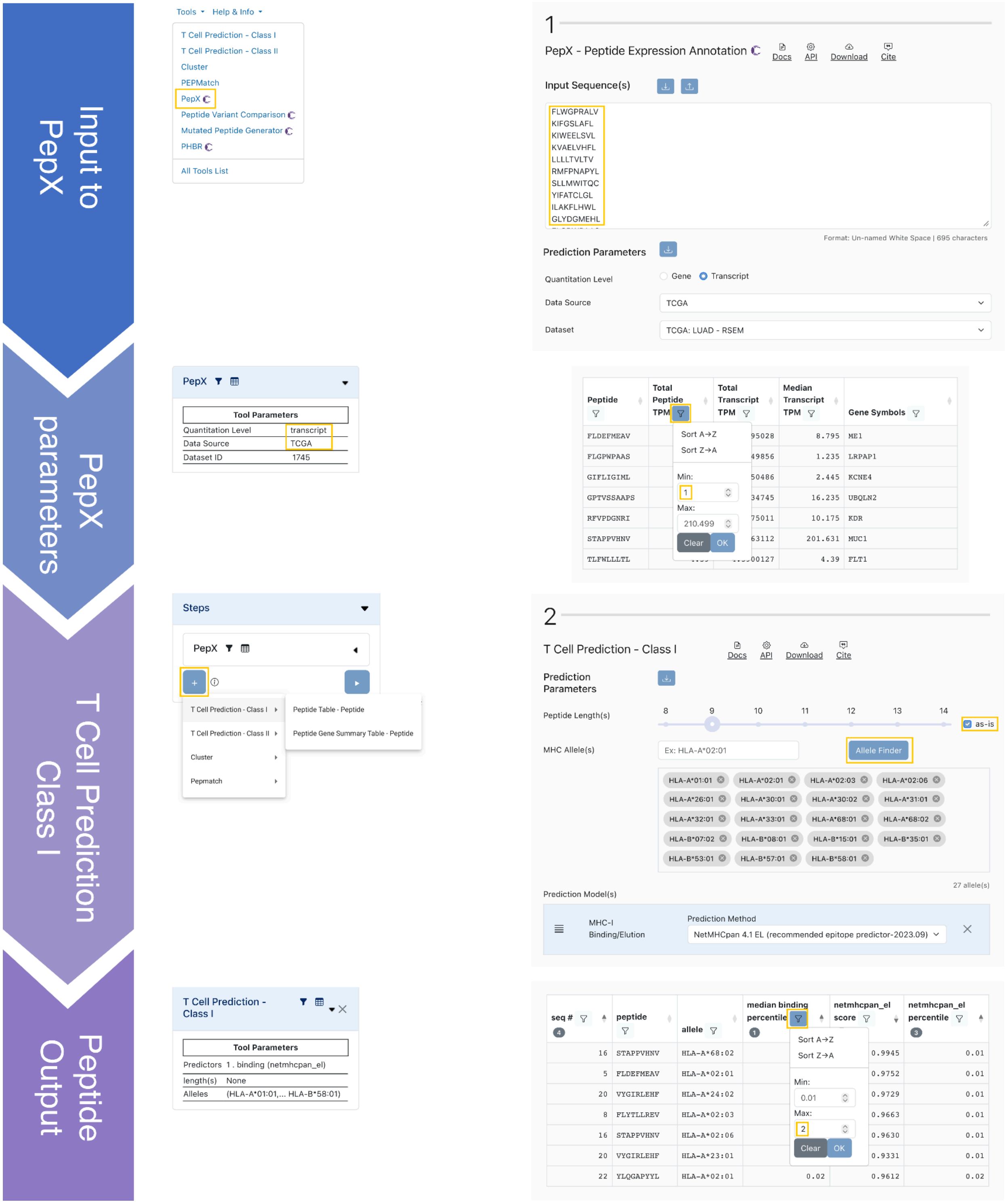

Supplementary Figure S1. Step-by-step walkthrough of Case Scenario I: Expression-based filtering of candidate tumor-associated antigens in NSCLC. Screenshots of the CEDAR NGT interface illustrating the sequential steps of the pipeline. (1) Input of candidate 9-11mer peptides derived from tumor-associated proteins into the PepX module, with selection of the TCGA-LUAD dataset and transcript-level quantification. (2) Filtering of peptides based on Total Transcript TPM values to retain candidates with confirmed expression in lung adenocarcinoma. (3) Configuration of the MHC class I prediction step using the NetMHCpan 4.1 EL model and the 27-allele panel covering more than 97% of the global population. (4) Filtering of results based on median binding percentile rank to retain strong and weak binders. The pipeline interface sidebar shows the sequential structure of the analysis and the parameters applied at each step.

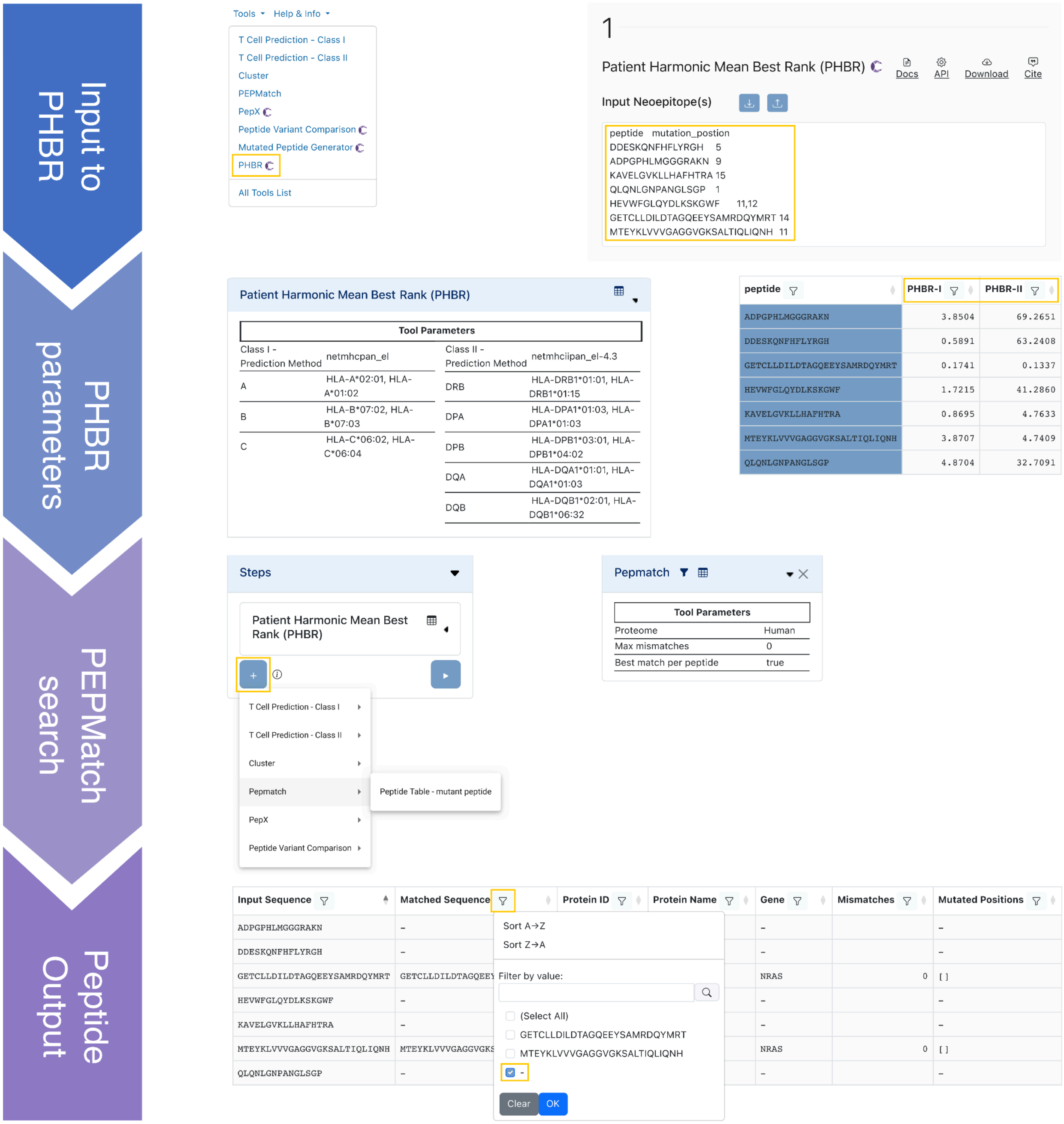

Supplementary Figure S2. Step-by-step walkthrough of Case Scenario II: Neoepitope discovery for personalized immunotherapy in glioblastoma. Screenshots of the CEDAR NGT interface illustrating the sequential steps of the pipeline. (1) Input of long mutated peptides derived from whole-exome sequencing data into the Patient Harmonic-mean Best Rank (PHBR) tool, with specification of the patient's MHC class I and class II alleles and selection of NetMHCpan 4.1 EL and NetMHCIIpan 4.3 EL prediction models. (2) Ranking and filtering of mutations based on PHBR scores to prioritize candidates with the highest likelihood of presentation across the patient's full HLA genotype. (3) Configuration of the PEPMatch module

for screening prioritized candidates against the human reference proteome with zero mismatches permitted. (4) Filtering of results to retain only peptides with no exact match in the human proteome, excluding sequences subject to central immune tolerance. The pipeline interface sidebar shows the sequential structure of the analysis and the parameters applied at each step.

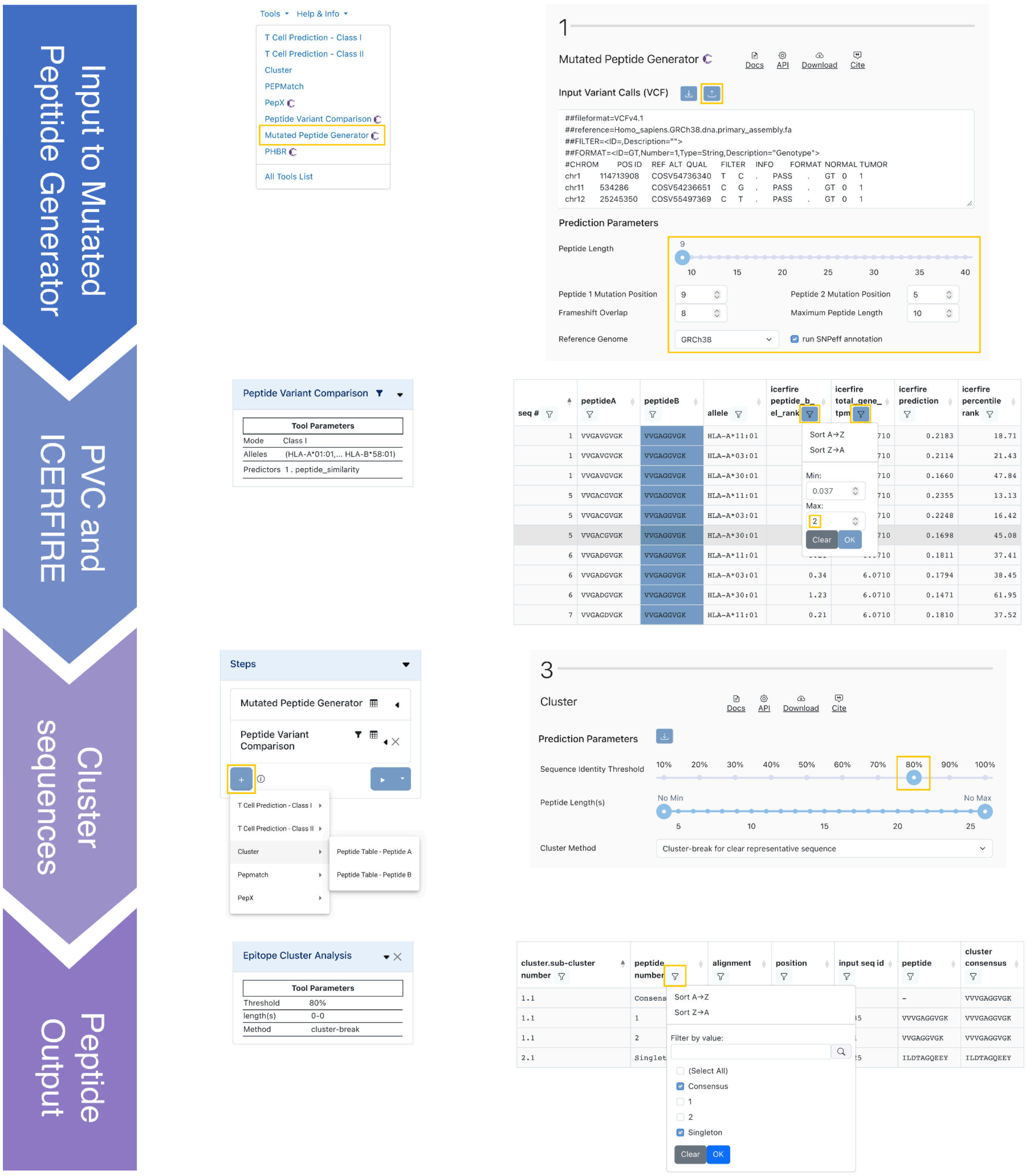

Supplementary Figure S3. Step-by-step walkthrough of Case Scenario III: Evaluation of shared RAS neoepitopes across tumor types. Screenshots of the CEDAR NGT interface illustrating the sequential steps of the pipeline. (1) Input of KRAS, NRAS, and HRAS hotspot mutations in VCF format into the Mutated Peptide Generator (MPG), for generation of 9mer neopeptides with mutations positioned at the C-terminal MHC anchor position and the central TCR contact site. (2) Configuration of the Peptide Variant Comparison (PVC) tool with the 27-allele panel and the ICERFIRE neoepitope immunogenicity model, with filtering based on eluted ligand percentile rank and TCGA pan-cancer expression values. (3) Application of the Clustering module to remove redundant candidates using an 80% sequence identity threshold. (4) Non-redundant representative set of broadly presentable pan-cancer neoepitope candidates. The pipeline interface sidebar shows the sequential structure of the analysis and the parameters applied at each step.
